# Proximal Row Carpectomy with Total Scapoidectomy vs. Conventional Carpal Resection for ReMotion Total Wrist Arthroplasty

**DOI:** 10.3390/jcm10091865

**Published:** 2021-04-26

**Authors:** Stefan M. Froschauer, Matthias Holzbauer, Dietmar Hager, Oskar Kwasny, Dominik Duscher

**Affiliations:** 1 Department for Trauma Surgery and Sport Traumatology, Med Campus III, Kepler University Hospital Linz, Krankenhausstrasse 3, 4020 Linz, Austria; Oskar.kwasny@kepleruniklinikum.at; 2 Faculty of Medicine, Johannes Kepler University Linz, Altenbergerstraße 69, 4020 Linz, Austria; dominikduscher@me.com; 3 Microsurgical Training and Research Center (MAZ), Kepler University Hospital GmbH, Krankenhausstrasse 3, 4020 Linz, Austria; 4 Diakonissen Clinic Linz, Weißenwolffstrasse 15, 4020 Linz, Austria; ordination@dr-hager.at; 5 Department of Plastic, Reconstructive, Hand and Burn Surgery, BG-Trauma Center, Eberhard Karls University Tuebigen, Schnarrenbergstrasse 95, 72076 Tuebingen, Germany

**Keywords:** proximal row carpectomy, radial impaction syndrome, ReMotion prosthesis, surgical technique, total wrist arthroplasty

## Abstract

High complication rates in total wrist arthroplasty (TWA) still lead to controversy in the medical literature, and novel methods for complication reduction are warranted. In the present retrospective cohort study, we compare the outcomes of the proximal row carpectomy (PRC) method including total scaphoidectomy (*n* = 22) to the manufacturer’s conventional carpal resection (CCR) technique, which retains the distal pole of the scaphoid (*n* = 25), for ReMotion prosthesis implantation in non-rheumatoid patients. Mean follow-up was 65.8 ± 19.8 and 80.0 ± 28.7 months, respectively. Pre- and postoperative clinical assessment included wrist flexion-extension and radial-ulnar deviation; Disability of Arm, Shoulder, and Hand scores; and pain via visual analogue scale. At final follow-up, grip strength and satisfaction were evaluated. All complications, re-operations, and revision surgeries were noted. Clinical complications were significantly lower in the PRC group (*p* = 0.010). Radial impaction was detected as the most frequent complication in the CCR group (*n* = 10), while no PRC patients suffered from this complication (*p* = 0.0008). Clinical assessment, grip strength measurements, and the log rank test evaluating the re-operation as well as revision function showed no significant difference. All functional parameters significantly improved compared to preoperative values in both cohorts. In conclusion, we strongly recommend PRC for ReMotion prosthesis implantation.

## 1. Introduction

The first prostheses for total wrist arthroplasty (TWA) were developed in the 1960s, while the ReMotion prosthesis, with its release in 2002, is one of the most recent implant types [1]. Since then, many authors have reported data that are not conclusive. The main argument for prosthetic treatment is a considerable gain of range of motion, while critics cite prior studies reporting high complication and revision rates [2,3]. However, most studies containing outcomes of TWA were conducted on rheumatoid patients, because rheumatoid arthritis initially represented the major indication for this procedure. Nowadays, TWA is increasingly used for non-rheumatoid indications. Gupta, one of the design surgeons of the ReMotion prosthesis, already emphasized in his original surgical description that “osteoarthritis is a good indication”, and TWA “surpasses that of 4-corner fusion and scaphoid excision” in posttraumatic arthritis [1]. Regarding ReMotion TWA, the overall perception and potential drawbacks discussed in the literature might be caused by the predominance of rheumatoid patients. Thus, there are few articles [4,5,6] involving results of subcohorts with non-rheumatoid patients, while our previous study is—to the best of our knowledge—the first study solely including patients suffering from primary and posttraumatic arthritis.

Besides the discussion regarding proper indications for TWA based on different patients’ primary condition, age, bone stock, and activity levels, the present study aims to examine an alternative surgical technique for ReMotion TWA. The rationale for this approach was the fact that we have observed radial impaction syndrome as the most frequent postoperative complication in our previous study [7]. This represents an impingement between the prosthesis’s radial socket and the scaphoid. Affected patients describe pain (especially during radial deviation), swelling, redness, and tenderness in the radial wrist region. We hypothesized that this complication is caused by the distal pole of the scaphoid, which remains in situ while performing the manufacturer’s conventional carpal resection (CCR) technique. This resection implies removal of the lunate, triquetrum, proximal scaphoid, and the proximal cortex of the capitate [1]. Hence, we have developed a modified surgical method using proximal row carpectomy (PRC) including total scaphoidectomy without the manufacturer’s carpal cutting guide. This method intends to avoid the prosthesis’s radial socket impinging on carpal bones, thus preventing radial impaction syndrome.

The present study aims to compare CCR with the PRC technique for ReMotion prosthesis implantation in non-rheumatoid patients. Besides assessing the clinical complication rate, which represents our primary outcome parameter, this study evaluates any differences between both implantation techniques in terms of DASH scores, pain levels, range of motion, grip strength, reoperation, and revision rate.

## 2. Materials and Methods

Investigation of the hypothesis that PRC used for carpal prosthesis fixation results in a lower overall complication rate compared to the CCR method (using a retrospective cohort study comparing PRC with CCR for ReMotion TWA) was conducted. A retrospective review identified all patients who underwent TWA between July 2007 and February 2018 at a single institution. Compared to our previous study [7], we have extended the review period to identify all patients with a theoretical follow-up period of a minimum of 24 months. In this period, two level IV surgeons [8] performed TWA using CCR according to the manufacturer‘s surgical technique [1]. By contrast, one level IV surgeon applied the implantation technique including PRC.

We included all patients who received TWA using the ReMotion Total Wrist System (Stryker, Kalamazoo, MI, USA) due to the following non-rheumatoid indications: idiopathic osteoarthritis (OA), posttraumatic OA, scapholunate advanced collapse (SLAC) wrist, scaphoid nonunion advanced collapse (SNAC) wrist, and Kienbock’s and Preiser’s diseases. A follow-up period of more than 24 months was defined as another inclusion criterion. Moreover, due to the intention-to-treat design of this study, all pre- and postoperative clinical and radiographical parameters (see Section 2.2.) had to be available at our final examination.

Clinical complication rate was defined as the primary outcome variable of the present study.

In total, 56 TWAs (55 patients) matched the inclusion criteria and were invited for a follow-up examination. Seven patients did not follow up on this invitation, and 2 patients died of unrelated causes. Hence, 16% patients were lost to follow-up.

Finally, 21 patients (22 wrists) and 27 patients (27 wrists) could be allocated to the PRC and CCR study cohort, respectively. Patient demographics are displayed in Table 1, while indications are listed in Table 2.

The study was conducted according to the guidelines of the Declaration of Helsinki and approved by the Institutional Review Board (Ethics Commission of Johannes Kepler University Linz #1082/2021). Written informed consent was obtained from all subjects involved in the study.

### 2.1. Surgical Technique

**Figure 1:**
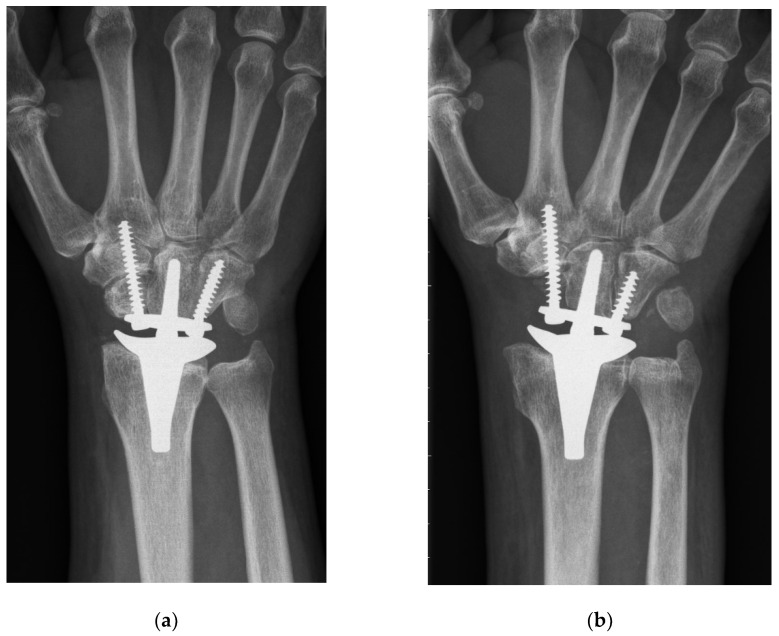
Postoperative radiograph showing both applied implantation techniques: ReMotion implantation using (**a**) CCR (conventional carpal resection) and (**b**) PRC (proximal row carpectomy). The results merely differ in the distal pole of the scaphoid being removed in the PRC cohort.

Both techniques implied a dorsal midline approach aligned with the third metacarpal. Next, the extensor retinaculum was divided obliquely over the third and fourth extensor compartment, and the joint capsule was opened by an open book incision. The CCR technique involved a manufacturer’s carpal cutting guide, which was aligned with the third metacarpal. Typically, the carpal resection included a resection of the lunate, triquetrum, proximal scaphoid, and the proximal cortex of the capitate, resulting in a cut perpendicular to the long axis of the radius (Figure 1a). On the contrary, the PRC method involved a sharp resection of the whole scaphoid, in addition to the prior mentioned bones, without using the carpal cutting guide. Moreover, the proximal part of the capitate and the tip of the hamate were resected using an oscillating saw, which was aligned perpendicularly to the long axis of the third metacarpal and strictly in the sagittal or dorsopalmar plane (Figure 1b).

Afterwards, the surgeries of both cohorts continued in the same way: the lunate and scaphoid fossa were contoured, removing the central ridge. A threaded Kirschner wire was aligned to the long axis of the radius in two planes via fluoroscopy. It was inserted in the medullary canal to mark the proper entry point and direction for the cannulated broaches. Following, the suitable size of the radial prosthesis was determined using preoperative templating and intraoperative size matching. Care was taken that the ulnar socket did not protrude beyond the distal radio-ulnar joint; hence, the triangular fibrocartilage complex could be preserved. The choice of size was not affected by the surgical technique. Radial preparation was finished by performing radial styloidectomy. Next, the capitate was prepared for the carpal implant by the carpal broach. A hole was predrilled towards the hamate and another one towards trapezoid and second metacarpal. After trail replacement, the radial and carpal prosthesis components were implanted press-fit without the use of cement, and the carpal prosthesis was fixated via two self-taping bone screws. Finally, the polyethylene carpal ball was snapped into place. After stability, as well as range of motion, were assessed, standard wound closure was performed.

### 2.2. Clinical and Radiographical Evaluation

Preoperatively, active range of motion (ROM) measurements included flexion, extension, and radial and ulnar deviation. Moreover, pain was assessed using a visual analogue scale (VAS) ranging from 0 (no pain) to 10 (worst imaginable pain). All patients completed the Disabilities of Arm, Shoulder, and Hand (DASH) questionnaire.

At the final follow-up examination, these assessments were repeated. Additionally, grip strength measurement of both hands was performed. Furthermore, patient satisfaction was evaluated while asking whether the patients would undergo the surgery again.

All occurring clinical complications at any follow-up time were noted, and the final complication rate was calculated for each cohort. Moreover, every reoperation and revision surgery, including the postoperative time they were performed, was assessed.

Final radiographs were screened for clinically inapparent abnormalities, including prosthesis migration, tilting, subsidence, screw breakage, and massive osteophytes, as well as radial and carpal radiolucency.

### 2.3. Statistical Methods

The arc of ROM was calculated for each plane by adding up flexion and extension angles, as well as radial and ulnar deviation measurement values. The difference in grip strength was assessed via subtracting grip strength of the operated hand from the healthy hand (The bilateral case was excluded from this calculation).

The Kolmogorov–Smirnov test was used to assess the outcome data distribution within every cohort. In case of normal data, outcomes were presented as mean ± standard deviation, while non-normal data were displayed as median (interquartile range).

Comparative testing between PRC and CCR cohorts was performed using the independent *t*-test or Mann–Whitney U-Test. Differences between the two groups in dichotomous and nominal variables were analyzed with the Chi-squared test or the Fisher’s exact test in case any value was <5.

Pre- and postoperative values within both cohorts were compared via the dependent Student’s *t*-test and Wilcoxon test depending on the data distribution.

For the primary outcome variable (i.e., clinical complication rate), the odds ratio including the 95% confidence interval was calculated. Moreover, a post hoc power analysis using G-power was conducted based on this parameter.

The cumulative revision-free and reoperation-free survival within every cohort was displayed using the Kaplan–Meier plot. The log rank test was applied to detect any differences between these functions.

A *p*-value smaller than 0.05 was considered as significant.

## 3. Results

According to statistical testing, the patient demographics parameters (Table 1) and indications (Table 2) are homogenously distributed in both cohorts.

Outcomes are summarized in Table 3, including a statistical comparison between both cohorts. Table 4 displays a longitudinal statistical work-up of clinical outcome parameters displaying significant improvements of every parameter in both cohorts.

Within the PRC cohort, we could detect five clinical complications in five patients. One patient presented with deep wound infection leading to wound irrigation, vacuum assisted closure, and final distally pediculated radial flap coverage. One patient developed insufficiency of the extensor retinaculum; thus, the polyethylene carpal ball was changed to a larger size. One patient presented with continuous pain in the carpus; massive, periprothetic radiolucency; and subsidence of the carpal prosthesis part. Consecutively, the loose prosthesis was explanted, and the therapeutic regimen was changed to arthrodesis using autologous iliac bone graft and plate osteosynthesis. One case of distal radioulnar joint pain was solved via resecting the head of the ulna. Another patient presented with massive pain and limited ROM, while radiographs showed massive osteophytes. This complication was addressed via removing osteophytes and performing arthrolysis. Moreover, clinically inapparent, radiographic abnormalities were observed in 12 patients: radial radiolucency (*n* = 8), carpal radiolucency (*n* = 6), screw breakage (*n* = 2), and extensive osteophytes (*n* = 1).

**Figure 2:**
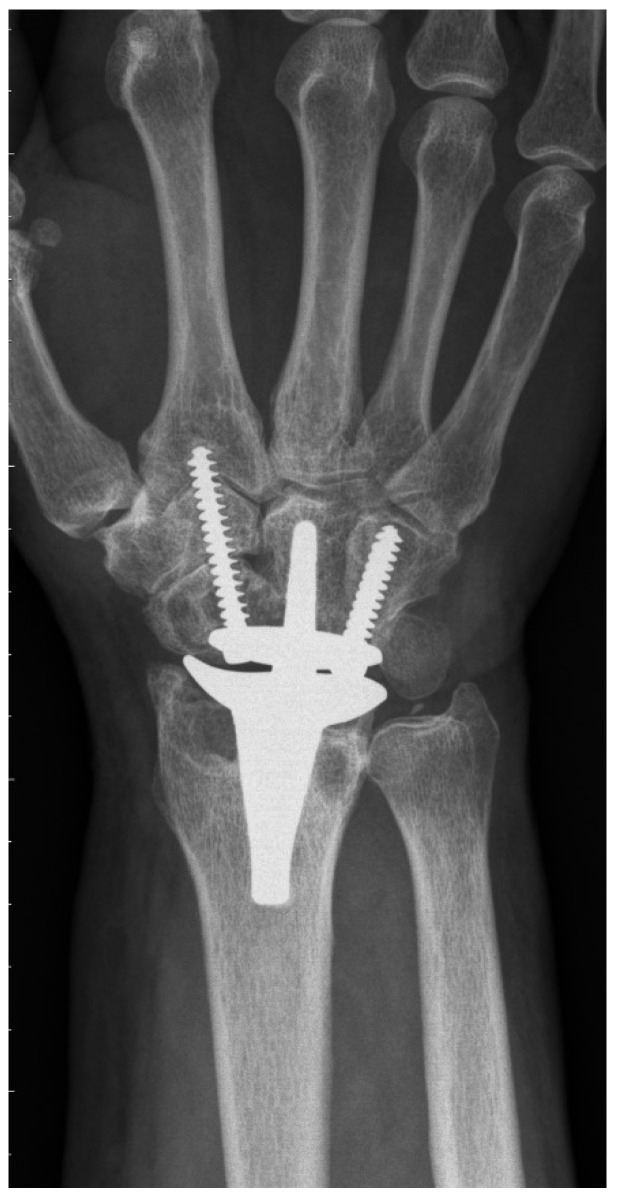
Final radiograph (72 months postoperatively) of a CCR cohort patient with massive radial impaction syndrome.

The CRR group showed 17 complications in 15 wrists. The most common one was radial impaction (*n* = 10) (Figure 2), leading to four re-operations including scaphoidectomy. Moreover, we could detect two ganglions, one complex regional pain syndrome, and one postoperative surgical site infection, which could successfully be treated with antibiotics. One patient received De Quervain’s tenosynovitis release. One patient presented with continuous pain in the region of the hamate with simultaneous radiolucency around the ulnar screw. During revision surgery, the loose ulnar screw was exchanged to a longer one. Another patient developed massive pain due to radial screw breakage, which was treated by exchanging the broken screw. Radiological abnormalities were observed in 19 patients: radial radiolucency (*n* = 19), carpal radiolucency (*n* = 14), and screw breakage (*n* = 1).

Finally, the odds of suffering a clinical complication were 0.20 (0.06–0.70) in the PRC cohort as opposed to PRC group.

Post hoc power analysis based on the primary outcome parameter (i.e., the clinical complication rate) revealed a value of 0.751.

**Figure 3:**
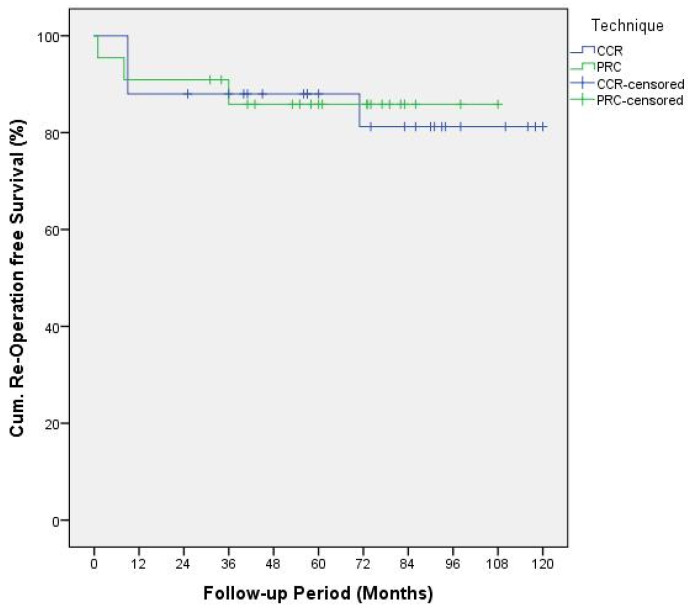
Kaplan–Meier Plot displaying cumulative re-operation-free survival functions.

**Figure 4:**
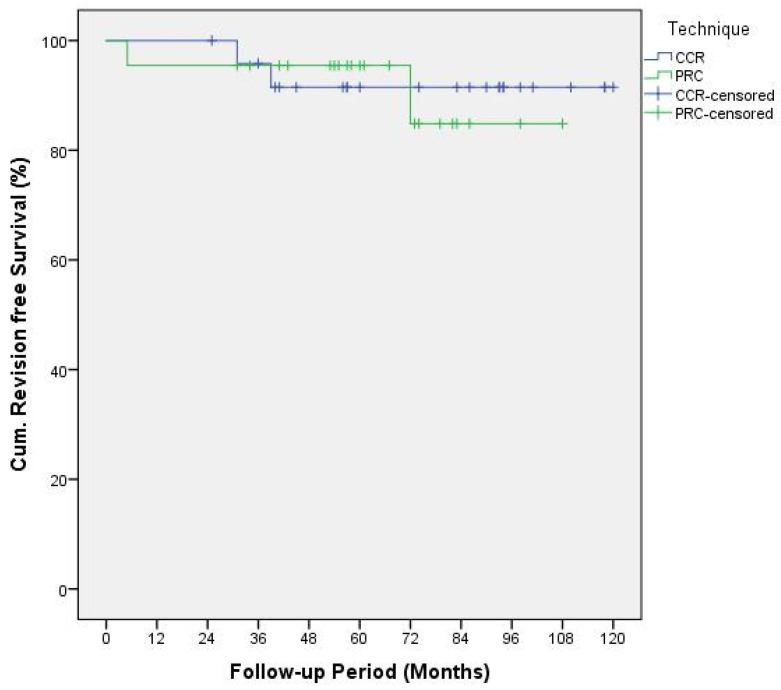
Kaplan–Meier Plot displaying cumulative revision-free survival functions.

Kaplan–Meier plots of the reoperation functions (Figure 3) and revision functions (Figure 4) were analyzed using log rank test resulting in *p*-values of 0.91 and 0.83, respectively.

## 4. Discussion

Among non-rheumatoid patients treated with ReMotion TWA, we found that the PRC implantation technique could significantly reduce the clinical complication rate compared to the CCR method. Hence, the odds of suffering from any complication were 80% lower if the distal pole of the scaphoid was removed (i.e., total scaphoidectomy). This finding can largely be explained by the significant reduction of the radial impaction syndrome’s occurrence to zero in the PRC cohort. Thus, we were able to verify the hypothesis of the present study.

Moreover, the ReMotion implantation technique had no significant influence on postoperative DASH scores, pain levels, ROM, grip strength measurements, re-operation, or revision surgery rates. Therefore, the PRC method for TWA resulted in no adverse effect. Especially, the absent distal pole of the scaphoid does not impair the stability of the carpal prosthesis. Although the radial fixation screw’s threads have higher bone contact, if the distal pole of the scaphoid remains in situ (CCR cohort), we found the same number of cases presenting with radial screw breakages (*n* = 2) in both cohorts.

To the best of our knowledge, PRC has never been previously described for primary TWA implantation in the current literature (see Table 5 for a summary of the literature). Gupta merely described, in his original surgical technique for ReMotion prosthesis, a salvage procedure for failed PRC or failed 4-corner fusions using TWA [1]. Interestingly, he recommended harvesting the pisiform for reconstruction of the missing distal pole of the scaphoid, where the radial screw is consecutively inserted. The present study shows that this additional step is not necessary, because fixation of the radial screw in the trapezoid and second metacarpal is sufficient for applying slight compression and rotational stability to the carpal prosthesis. Conney et al. [9] are the only authors who reported details on their carpal resection technique (i.e., the manufacturer’s recommended technique). Interestingly, we also found in the manufacturer’s operative technique that the producer recommends a more distal resection in patients with excessive carpal erosion or advanced degenerative joint disease [10]. However, we assume that this adaption would probably also not prevent TWA patients from being prone to suffering from radial impaction syndrome. This predominant clinical complication in the CCR group is most probably caused by the remaining distal pole of the scaphoid. Radiographically, this issue can be perfectly illustrated: Figure 2 shows a dorsopalmar radiograph of the wrist in neutral position with respect to radial and ulnar deviation (the third metacarpal and the long axis of the metacarpal are perfectly aligned). Even in this neutral position—without actively radially deviating the carpus—one can see contact or impingement between the prosthesis’s radial socket and the distal pole of the scaphoid. This situation led to four reoperations including resection of the distal pole of the scaphoid, which completely resolved the patient’s complaints. This further confirms the hypothesis of a correlation between the CCR technique and radial impaction syndrome. Moreover, we suppose that this complication is due to particular challenges associated with TWA. Inspecting the postoperative radiograph in Figure 1a, due to a sufficient distance between the socket and the scaphoid, the before-mentioned issue of radial impaction syndrome would be difficult to comprehend or predict. Despite intraoperative visual and fluoroscopic evaluation for any impingement in every extreme position while performing the CCR technique, radial impaction syndrome seems to develop in the postoperative course. One of the main reasons might be that TWA is very prone to periprosthetic, osseous remodeling. Osteophytes and osteolysis might cause or aggravate the morphologic correlate of radial impaction syndrome, which can be seen in Figure 2. This 6-year postoperative radiograph was taken from the same patient as in Figure 1a. The phenomenon has already been observed by Boeckstyns and Herzberg [11], who discuss possible reasons for osseous reactions in TWA. This publication mainly focused on radiolucency, which also represented a clinically asymptomatic, radiographic abnormality in both our cohorts.

Historically, most studies reporting TWA outcomes were conducted on patients with rheumatoid arthritis [3]. Recently, some authors have extended the range of indications for TWA to non-rheumatoid conditions and compared their results to rheumatoid arthritis [4,5,6]. The present study and the previously published study [7] are the first ones to solely present outcomes of non-rheumatoid patients.

To shed a comparative light on ReMotion prosthesis, Table 5 contains a review of the current literature containing all previous articles reporting about this implant type. Thus, we could detect that our cohorts could achieve the lowest postoperative DASH scores compared to all other articles. Moreover, both cohorts showed similar, satisfying ROM results compared to other studies involving non-rheumatoid patients [4,5].

There are some limitations of the present study. First, our cohorts include relatively few patients. Due to the retrospective design of this study, the allocation to one of the two cohorts was not performed via randomization, and the follow-up periods vary slightly. However, the post hoc power analysis based on the primary outcome variable revealed a satisfactory value. Moreover, only mid-term findings can be derived from our follow-up period. Further long-term randomized-control trials have to confirm the favorable results of this PRC technique, and might also study the effect on rheumatoid patients.

## 5. Conclusions

The present study is reporting about PRC for ReMotion prosthesis implantation. The study’s hypothesis was confirmed: removing the distal pole of the scaphoid (PRC technique) can significantly reduce radial impaction syndrome and the overall complication rate, while no adverse effects—especially loosening of carpal screws—could be detected.

Therefore, we strongly recommend the PRC method for ReMotion prosthesis implantation.

## Figures and Tables

**Table 1:** Patient Demographics.

	PRC	CCR	*p*-Value
Patients	21	25	-
Wrists	22	25	-
Age	59.3 ± 11.5	56.9 ± 11.3	0.47 ^1^
Sex (f/m)	16/6	15/10	0.36 ^2^
Side (l/r)	12/10	17/8	0.34 ^2^
Follow-up (months)	65.8 ± 19.8	80.0 ± 28.7	0.055 ^1^

Statistical testing was performed using ^1^ unpaired Student’s *t*-test and ^2^ Chi-squared test. PRC: Proximal Row Carpectomy; CCR: Conventional Carpal Resection.

**Table 2:** Indications for TWA (Total wrist arthroplasty) implantation.

	PRC	CCR	*p*-Value
Idiopathic OA	6	8	0.72 ^1^
Posttraumatic OA	3	4	1.0 ^2^
SLAC wrist	5	7	0.68 ^1^
SNAC wrist	6	4	0.48 ^2^
Kienbock’s disease	1	2	1.0 ^2^
Preiser’s disease	1	0	1.0 ^2^

Statistical testing was performed using ^1^ Chi-squared test and ^2^ Fisher’s exact test. OA: Osteoarthritis; SLAC: Scapho-lunate advanced Collpas; SNAC: Scophoid Non-Union advanced collaps.

**Table 3:** Outcome parameters.

Parameter	Time	PRC	CCR	*p*-Value
		(*n* = 22)	(*n* = 25)	
DASH scores	preop.	65.6 ± 13.7	61.8 ± 11.9	0.32 ^1^
	postop.	32.5 ± 23.4	27.3 ± 23.8	0.46 ^1^
VAS for pain	preop.	7 (1.3)	7 (1.5)	0.11 ^2^
	postop.	3 (3.5)	2 (3.0)	0.52 ^2^
Flexion	preop.	25° (11°)	30° (13°)	0.94 ^2^
	postop.	35° (10°)	35° (10°)	0.53 ^2^
Extension	preop.	20° (9°)	20° (8°)	0.26 ^2^
	postop.	31° ± 10°	32° ± 10°	0.77 ^1^
Arc of ROM (Flex. + Ext.)	preop.	43° ± 16°	45° ± 16°	0.58 ^1^
	postop.	65° (20°)	70° (25°)	0.46 ^2^
Radial deviation	preop.	10° (5°)	5° (5°)	0.20 ^2^
	postop.	15° (10°)	10° (13°)	0.06 ^2^
Ulnar deviation	preop.	15° (5°)	15° (5°)	0.010 ^2^
	postop.	20° (10°)	30° (10°)	0.35 ^2^
Arc of ROM (Rad. + Uln. dev.)	preop.	25° (11°)	20° (10°)	0.025 ^2^
	postop.	39° ± 10°	37° ± 11°	0.61 ^1^
Grip strength operated hand	postop.	24.1 ± 9.9	29.2 ± 12.8	0.17 ^1^
Grip strength healthy hand	postop.	34.4 ± 14.7	34.6 ± 13.6	1.0 ^1^
Difference in grip strength	postop.	10.0 ± 13.6	5.4 ± 9.8	0.23 ^1^
Satisfied	postop.	20 (91%)	24 (96%)	0.59 ^3^
Clinical complication	postop.	5 (23%)	15 (60%)	0.010 ^4^
Radial impaction	postop.	0 (0%)	10 (40%)	0.0008 ^3^
Radiological abnormality	postop.	12 (54%)	19 (76%)	0.12 ^4^
Reoperation	postop.	3 (14%)	4 (16%)	1.0 ^3^
Revision	postop.	2 (9%)	2 (8%)	1.0 ^3^

Statistical testing was performed using ^1^ unpaired Student’s *t*-test, ^2^ Mann–Whitney U-test, ^3^ Fisher’s exact test, and ^4^ Chi-squared test; preop. = preoperative; postop. = postoperative. VAS: Visual analog scala; ROM: Range of Motion.

**Table 4:** *P*-values resulting from the comparison between pre- and postoperative outcomes within every group.

	PRC	CCR
DASH scores	<0.0001 ^1^	<0.0001 ^1^
VAS for pain	<0.0001 ^2^	<0.0001 ^2^
Flexion	0.0002 ^2^	0.002 ^2^
Extension	0.0004 ^2^	0.0006 ^2^
Arc of ROM (Flex. + Ext.)	<0.0001 ^1^	0.0003 ^2^
Radial deviation	0.012 ^2^	0.0006 ^2^
Ulnar deviation	<0.0001 ^2^	0.0006 ^2^
Arc of ROM (Rad. + Uln. dev.)	<0.0001 ^2^	<0.0001 ^2^

Statistical testing was performed using ^1^ paired Student’s *t*-test and ^2^ Wilcoxon test.

**Table 5:** Review of the current literature involving all publications reporting about ReMotion prosthesis.

Authors (Publication)	Patients (n)	Follow-Up (Months)	Indications	DASH Score	VAS	Flexion	Extension	Radial Deviation	Ulnar Deviation	Survival Rate	Revision Rate
Cooney et al. (2012) [9]	22	72 (42–180) ^a^	36 RA 10 non-RA ^a^	37	2.3	29.9°	40°	7.7°	20.5°	6y: 97%	n/a
Herzberg et al. (2012) [5]	215	48 (24–96)	129 RA	−20 ^b^	−4.8 ^b^	29°	29°	5°	24°	8y: 92%	6/129
			86 non-RA	−21 ^b^	−5.4 ^b^	37°	36°	10°	28°	8y: 94%	5/86
Bidwai et al. (2013) [12]	13	33 (14–56)	13 RA	n/a	3.2	35°	23°	7°	15°	n/a	1/13
Boeckstyns and Herzberg (2014) [11]	65	78 (60–108)	50 RA	41	2.9	25°	28°	6°	20°	9y: 90%	6/65
			15 non-RA	50	2.3	44°	43°	7°	28		
Sagerfors et al. (2015) [13]	87	84 ± 30	68 RA–19 non-RA	−12 ^b^	Rest: −2 Activity: −5.0 ^b^	0° ^b^	5° ^b^	0° ^b^	0° ^b^	8y: 94%	n/a
Chevrollier et al. (2016) [6]	7	36 (13–60)	2 RA	30; 27; ^b^	2; 1; ^b^	ROM: 25°; 57°; ^c^		ROM: 13°; 28°; ^c^		100%	0/2
			5 non-RA	36 ± 19	2.6 ± 3.0	ROM: 30° ± 10°		ROM: 22° ± 12°		100%	1/5
Froschauer et al. (2019) [7]	39	84 (36–144)	39 non-RA	29 (26)	2 (1.8)	40° (9°)	35° (10°)	15° (4°)	30° (9°)	97%	1/39
Honecker et al. (2019) [14]	23	69 (12–124)	19 R–4 non-RA	−18.9	−4.4	5.3	10.8	n/a	n/a	6y: 91%	4/23
Fischer et al. (2020) [15]	69	120	57 RA–12 non-RA	−14 ^b^	Rest: −1.5 Activity: −5 ^b^	0 ^b^	5 ^b^	5 ^b^	0 ^b^	94%	4/69

^a^ Only results of all cohorts (i.e., containing other prosthesis types) are reported; ^b^ difference between final follow-up values compared to the preoperative ones; ^c^ singe values are given; RA = rheumatoid arthritis; ROM = range of motion.

## Data Availability

The data that support the findings of this study are available from the first author (S.M.F.), upon reasonable request.
